# Endoscopic hand suturing of small intestine and colon: complete suturing of a post-endoscopic submucosal dissection mucosal defect at the anastomosis after right hemicolectomy

**DOI:** 10.1055/a-2304-8184

**Published:** 2024-06-05

**Authors:** Takuma Okamura, Tomonari Ikeda, Tatsuki Ichikawa, Kazuhiko Nakao

**Affiliations:** 1 13650 Department of Gastroenterology, Nagasaki Harbor Medical Center, Nagasaki, Japan; 2 200674 Department of Comprehensive Community Care Systems, Nagasaki University Graduate School of Biomedical Sciences, Nagasaki, Japan; 3 88380 Department of Gastroenterology and Hepatology, Nagasaki University Hospital, Nagasaki, Japan

Endoscopic hand suturing (EHS) was first reported by Goto et al. 1 . Although still in its infancy, it is expected to prevent complications, such as postoperative bleeding and perforation; however, due to the complexity of the procedure and the need to carry the needle to the target ulcer, it is currently used only in the distal colon and stomach 2 3 . Herein, we report the use of EHS at the anastomotic site after right hemicolectomy.

A 79-year-old man was referred to our hospital because of a nongranular-type laterally spreading tumor that had been noted at the postoperative anastomosis after right hemicolectomy ( Fig. 1 **a, b** ). We performed endoscopic submucosal dissection under texture and color enhancement imaging (TXI) and magnification using a GIF-XZ1200, with en bloc resection achieved in 88 minutes ( Fig. 1 **c, d** ). The mucosal defect was semicircumferential, with half of the defect occupying the small-intestinal side.

**Fig. 1:**
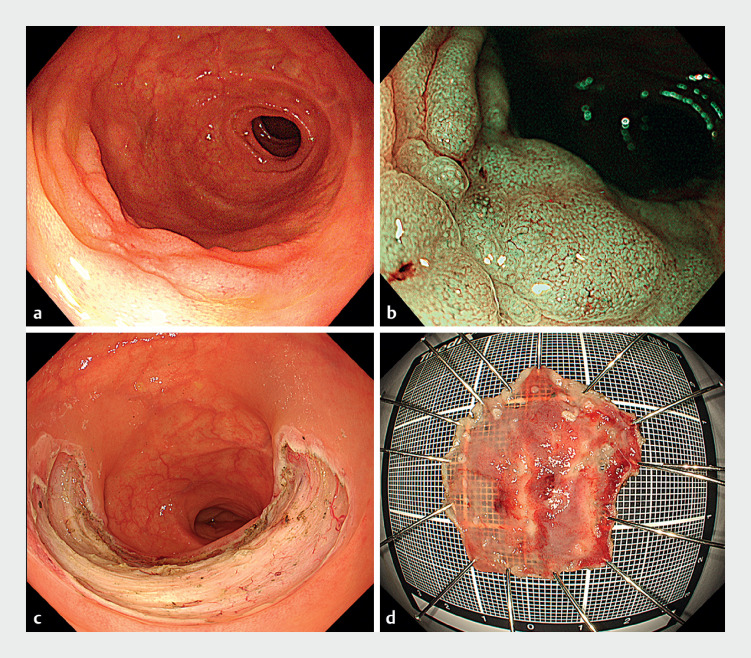
Images of the initial procedure to remove a nongranular-type laterally spreading tumor at the postoperative anastomosis: **a, b** before resection; **c, d** following endoscopic submucosal dissection showing: **a** the endoscopic appearance on white-light imaging; **b** the appearance on narrow-band imaging with magnification; **c** the endoscopic appearance of the mucosal defect; **d** the resected specimen, with the lesion removed en bloc.

Because the patient had diabetes mellitus and was taking antithrombotic medication, we needed to perform EHS, which is a secure and firm wound-closure method, using a wound-closure device (SutuArt, Olympus, Tokyo, Japan) and barbed suture (V-Lock, Medtronic, USA), to prevent postoperative bleeding and perforation ( Fig. 2 ). The needle was delivered through an overtube, grasping the tail of the needle with the wound-closure device, with the needle tip positioned inside the hood. Technically, complete closure was achieved, and no adverse events were reported ( Fig. 3 , Video 1 ). No evidence of wound dehiscence was observed at follow-up endoscopy the following day ( Fig. 4 ). The patient was able to resume eating the day after treatment and was discharged home on the second postoperative day, without experiencing any complications.

**Fig. 2:**
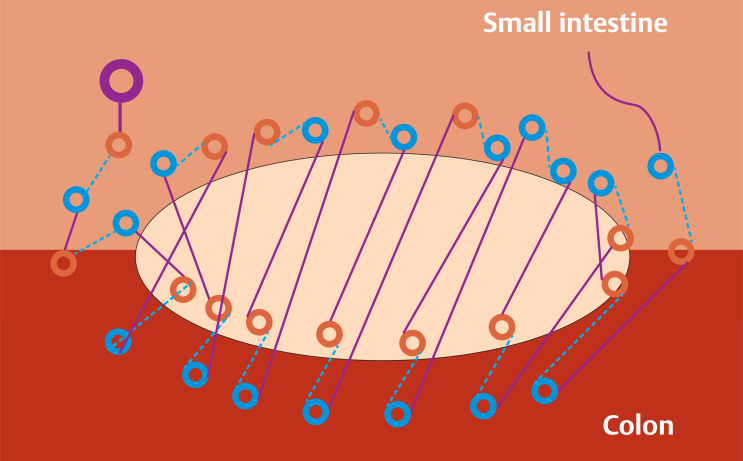
Schematic of the endoscopic closure of a mucosal defect at the anastomotic site, extending from the small intestine to the colon, using an innovative wound-closure device to suture in the longitudinal direction. Yellow circles, needle entries; blue circles, needle exits; light blue dotted lines, threads within submucosa; purple lines, threads on the mucosa.

**Fig. 3:**
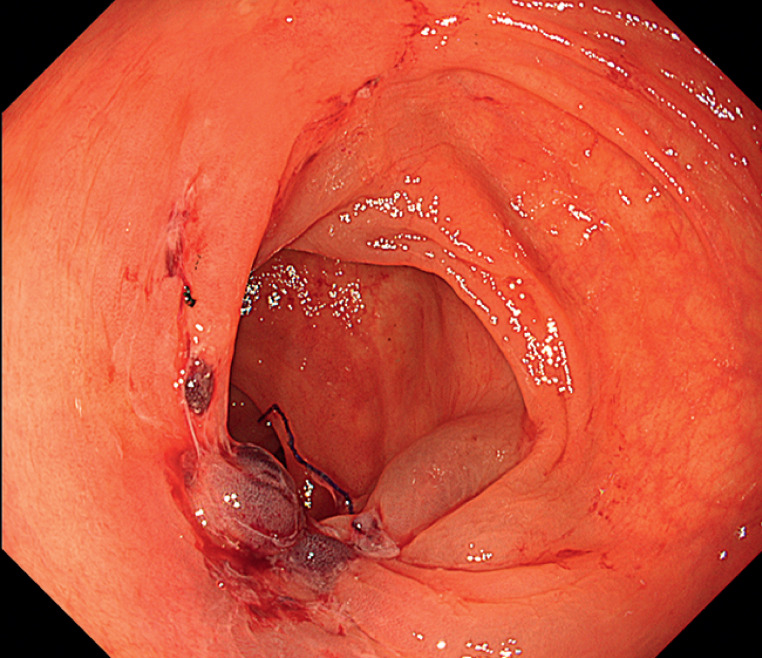
Endoscopic view of the anastomotic site after endoscopic hand suturing to close a defect extending across the small intestine and colon.

**Fig. 4:**
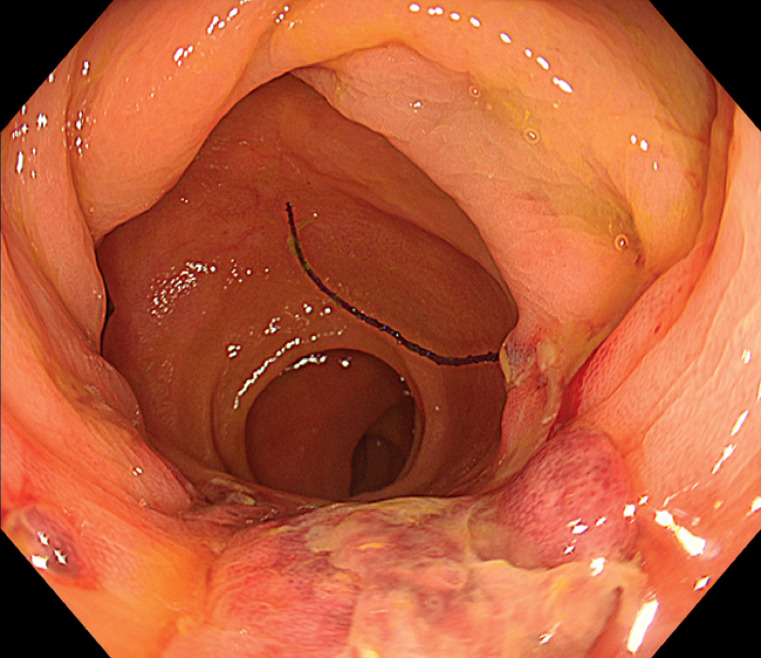
Endoscopic view on the day following the endoscopic hand-suturing procedure to close the defect across the small intestine and colon.

Endoscopic hand suturing of a mucosal defect at the postoperative anastomosis of the small and large intestines.Video 1

This is the first report of EHS of a mucosal defect at the postoperative anastomosis of the small intestine and colon. EHS could be added to the range of existing closure methods for mucosal defects at a postoperative anastomosis.

Endoscopy_UCTN_Code_TTT_1AO_2AO

## Notes

Endoscopy E-Videos https://eref.thieme.de/e-videos
*E-Videos* is an open access online section of the journal *Endoscopy* , reporting on interesting cases and new techniques in gastroenterological endoscopy. All papers include a high-quality video and are published with a Creative Commons CC-BY license. Endoscopy E-Videos qualify for HINARI discounts and waivers and eligibility is automatically checked during the submission process. We grant 100% waivers to articles whose corresponding authors are based in Group A countries and 50% waivers to those who are based in Group B countries as classified by Research4Life (see: https://www.research4life.org/access/eligibility/ ). This section has its own submission website at https://mc.manuscriptcentral.com/e-videos .
